# Goals of older hospitalized patients with multimorbidity

**DOI:** 10.1007/s41999-023-00746-5

**Published:** 2023-01-13

**Authors:** Sanne Tent, Marlies Verhoeff, Suzanne Festen, Barbara C. van Munster

**Affiliations:** 1 University of Groningen, University Medical Center Groningen, Department of Geriatric Medicine, University of Groningen, Hanzeplein 1, 9700 RB Groningen, The Netherlands; 2 Knowledge Institute of the Federation of Medical Specialists, Utrecht, The Netherlands

**Keywords:** Multimorbidity, Patient-centered healthcare, Goal setting, Older hospitalized patients

## Abstract

**Purpose:**

Patient-centered healthcare, with aligning treatment to a patients’ goal, is recognized by experts as essential to improve healthcare for older patients with multimorbidity. Little literature exists on goals of these specific patients. Therefore, we aimed to explore goals of older hospitalized patients with multimorbidity and compare their goals to those of older hospitalized patients without multimorbidity.

**Methods:**

Older hospitalized patients (aged ≥ 70 years) were included in a prospective mixed-methods cohort study at the University Medical Centre Groningen, the Netherlands. Goals were assessed by a standardized interview, whereafter they were categorized and analyzed descriptively.

**Results:**

Four hundred and ninety-three older hospitalized patients (median age 75 (IQR 72–80), 64% male) were included, of which 223 patients presented with multimorbidity (45%). Goals mentioned most often were ‘controlling disease’ and ‘alleviating complaints’. No differences were found in goals mentioned by patients with and without multimorbidity. Forty-one percent of both patients with and without multimorbidity mentioned goals that were disease-unrelated.

**Conclusion:**

No major differences were found in goals of older hospitalized patients with and without multimorbidity. However, the large proportion of patients mentioning disease-unrelated goals emphasizes the importance of goal elicitation by healthcare professionals within hospital care to provide optimally integrated care.

## Key Summary Points

**Aim:**

To explore goals of older hospitalized patients with multimorbidity and compare their goals to those of older hospitalized patients without multimorbidity.

**Findings:**

No differences were found in goals mentioned by patients with and without multimorbidity. Forty-one percent of both patients with and without multimorbidity mentioned goals that were disease-unrelated.

**Message:**

The large proportion of patients mentioning disease-unrelated goals emphasizes the importance of goal elicitation by healthcare professionals within hospital care to provide optimally integrated care.

## Introduction

Patients with multimorbidity generally experience a higher burden of disease, functional disabilities, reduced quality of life and increased mortality compared to patients with a single disease [1, 2]. Presence of multiple conditions results in more frequent physician visits, polypharmacy, hospitalizations, and higher total care expenditures [3]. For patients with multimorbidity, organization, coordination and compliance to a burden of regimens can be difficult [4]. Although multimorbidity is often associated with functional impairment and psychosocial problems, care is mostly disease oriented [5, 6].

To improve healthcare for older hospitalized patients with multimorbidity, expert consensus exists on the importance of implementing patient-centered care [7]. Patient-centered care is focused on the patient self, arranged from the patients’ perspective and the patient’s healthcare needs [8]. It was shown that patients receiving patient-centered healthcare choose less invasive treatment options and numbers of screening tests and their psychological health (e.g., depression, quality of life) and capability to self-manage a condition improved [9, 10].

An important aspect of patient-centered care for patients with multimorbidity is aligning healthcare to personal goals. Patient goals-aligned decision making is a continuous process of (1) establishing individual patient’s health goals, (2) starting, continuing, or stopping care based on these health goals and potential benefits versus harms or burden, and (3) aligning decisions and care for a patient between clinicians or other caregivers [11]. Patients’ goals are in particular helpful to guide clinical decisions when treatment options are interfering with each other [12].

From literature, we know older adults rate life enjoyment, social relations, quality of life, mobility and maintenance of autonomy as most important in daily life and the more chronic care setting [13–17]. Recently, remaining alive, feeling better and improving condition were established as important goals for older hospitalized patients [18]. However, it is unknown if during hospitalization, patients with and without multimorbidity pursue corresponding goals. Since patients with multimorbidity experience more burden of their disease, functional disabilities, and a reduced quality of life [1, 2], it might be that their goals while being hospitalized focus on preserving their level of daily functioning at home. By contrast, hospitalized patients who experience a single disease might focus more on treating their disease or accompanying complaints. In this study, we, therefore, aimed to explore goals of older hospitalized patients with multimorbidity and without multimorbidity to better align future hospital care.

## Methods

### Study design, setting and participants

This study was a prospective mixed-methods cohort study. Recruitment and inclusion of medical and surgical patients admitted to wards of the University Medical Centre Groningen (UMCG), the Netherlands, took place between February 2017 and March 2020. Inclusion criteria were: (1) aged 70 years and older, (2) a hospital stay for at least two consecutive days, and (3) understanding and ability to communicate in the Dutch spoken language. Patients with any (temporary) cognitive condition that could have influenced decision making (e.g., delirium and diagnosed dementia) prior to interview were excluded. On weekdays, electronical patient files of hospitalized older patients (for less than 96 h) were screened for eligibility by a research nurse and, if eligible, approached by trained researchers from the geriatric department. Data were collected during the first 96 h of hospitalization.

### Demographic characteristics

Patient characteristics during hospitalization were assessed by a face-to-face standardized interview conducted by a trained researcher to establish age, gender, living situation, marital status, and level of education. Level of education was categorized in low, middle and high according to definitions of Verhage [19]. Department of admission and admission type were collected from the Electronic Health Record (EHR).

### Geriatric assessment

Multimorbidity was defined as presenting with polypharmacy and two or more diseases scored with the Charlson Comorbidity index (CCI) [20]. The CCI (scored using the EHR) was used, since most relevant comorbidities that cause disease burden are present in this questionnaire. Polypharmacy was defined as the intake of five or more medicines. Patients were asked whether they had a fall in the last 6 months. The short nutritional assessment questionnaire (SNAQ) was conducted to assess if patients were malnourished (defined by a score of two or more) [21]. Problems with mobility and pain/discomfort were assessed by the EQ-5D-3L (a score of one or more on the corresponding domain) and self-rated health was assessed using the EQ-5D-VAS [22, 23]. Impairments in activities of daily living (ADL) were defined as a score of one or more on the Katz-15, an instrument that assesses a combination of scores on ADL and instrumental ADL [24]. Both the subdomains of the EQ-5D-3L and Katz-15 were based on health status 2 weeks before hospitalization. To assess the cognitive state of patients, the 6-item Cognitive Impairment Test (6-CIT) was conducted and categorized in normal cognition (score lower than eight) and cognitive impairment (score of eight or higher) [25, 26]. Presence of depression in the last month was screened using the two-item Patient Health Questionnaire (PHQ-2) (depression was defined as a score of one or more) [27]. During the geriatric assessment, we used validated Dutch versions or translations of instruments.

### Goal setting and coding

During the face-to-face standardized interview conducted by the trained researcher, goals of participants were assessed. Participants were asked: ‘What do you hope to accomplish with this hospitalization?’. The trained researcher was allowed ask probing questions to clarify answers and they verified with the patient if answers were written down correctly. Patients could mention multiple goals, which were written down individually. If multiple goals were written down within the first answer, the overall goal was selected. To illustrate, if a patient answered: “That I improve my fitness and can execute activities of daily living again, like the household and going to the store”, the goal is categorized in ‘improving daily functioning’ because “improving fitness” was needed to reach the overall goal “execute activities of daily living”.

For the current study, the first goal mentioned by the patient was analyzed. The patients’ goals were independently coded and categorized by two researchers (TvV and SB) using the method previously described by Van der Kluit et al. [28]. In this method, 36 codes were connected to the answer of the patient and thereafter subdivided into nine goal categories. Examples of individual quotes can be found in Table 1, with the corresponding goal categories. When a goal did not fit one of the nine goal categories, the goal was categorized as ‘undefinable’. After individual coding was completed, codes were compared. In case of disagreement, a third and independent researcher (SF) was consulted for a final decision until all data had been coded.

**Table 1:** Examples of individual quotes given by the older hospitalized patients, with corresponding goal categories

Category	Example quotes
Controlling disease	“To prevent my aneurysm from bursting” “To control my heart arrhythmias” “To heal the erysipelas”
Alleviating complaints	“To reduce the pain with the help of pain medication” “To reduce the presence of mucus, so that my breathing eases” “To reduce the complaints in my intestines”
Staying alive	“To extend my life with a couple of years” “That I can live for two or three more years” “To be able to make it to my 80th birthday”
Wanting to know what the matter is	“That they find the underlying cause of my oxygen deficiency” “That they find out why I am falling” “To get certainty on the spots in my lungs and intestines.”
Resuming work/hobbies	“To be able to work In the garden again” “To get back to work” “To work in the garden, take care of my dog and be active”
Improving condition	“To get my strength back, since walking is very laborious for me” “To get more energy” “To get the same level of physical fitness as one year ago”
Regain/maintain autonomy	“That after surgery I am able to function independently” “To be able to function the way it was before my surgery” “That I can pick up my old life again”
Improving daily functioning	“To be able to sit in a room, drink a cup of coffee and read the newspaper.” “To do the groceries by myself” “To be able to read a book, cook dinner and set the table”
Improving/maintaining social functioning	“To be able to babysit my grandchildren” “To go back home and live with my husband” “To be able to undertake activities with my husband”
Undefinable	“To leave as soon as possible” “To stop smoking” “Nothing. I want to go to the crematory”

Aside from the categorization into the ten goal categories, a division was made between disease-related goals and disease-unrelated goals. Where disease-related goals focus on the disease or complaints a patient is presenting, disease-unrelated goals focus on functioning outside the hospital in the broadest sense. Disease-related goals were ‘wanting to know what the matter is’, ‘controlling disease’, ‘staying alive’ and ‘alleviating complaints’. Goals categorized into ‘improving condition’ (fitness), ‘improving daily functioning’, ‘improving/maintaining social functioning’, ‘resuming work/hobbies’, ‘regain/maintain autonomy’ and ‘undefinable’ were defined as disease-unrelated goals.

### Statistical analysis

To determine baseline characteristics of the population, frequency counts of central tendency were conducted. To assess differences in patients with and without multimorbidity, independent t-tests, Mann–Whitney *U* tests and Chi^2^ tests were performed as appropriate. Goal categories were visualized for older hospitalized patients with and without multimorbidity and analyzed descriptively.

### Statement of ethics

The study was not subjected to the Dutch Medical Research Human Subjects Act (file number: 201600268) as confirmed by the Medical Research Ethics Committee. The study was approved by the local institutional review board. The study was conducted in accordance with the Declaration of Helsinki and Good Clinical Practice Guidelines. Written informed consent was obtained from all patients before inclusion in the study.

## Results

**Fig. 1:**
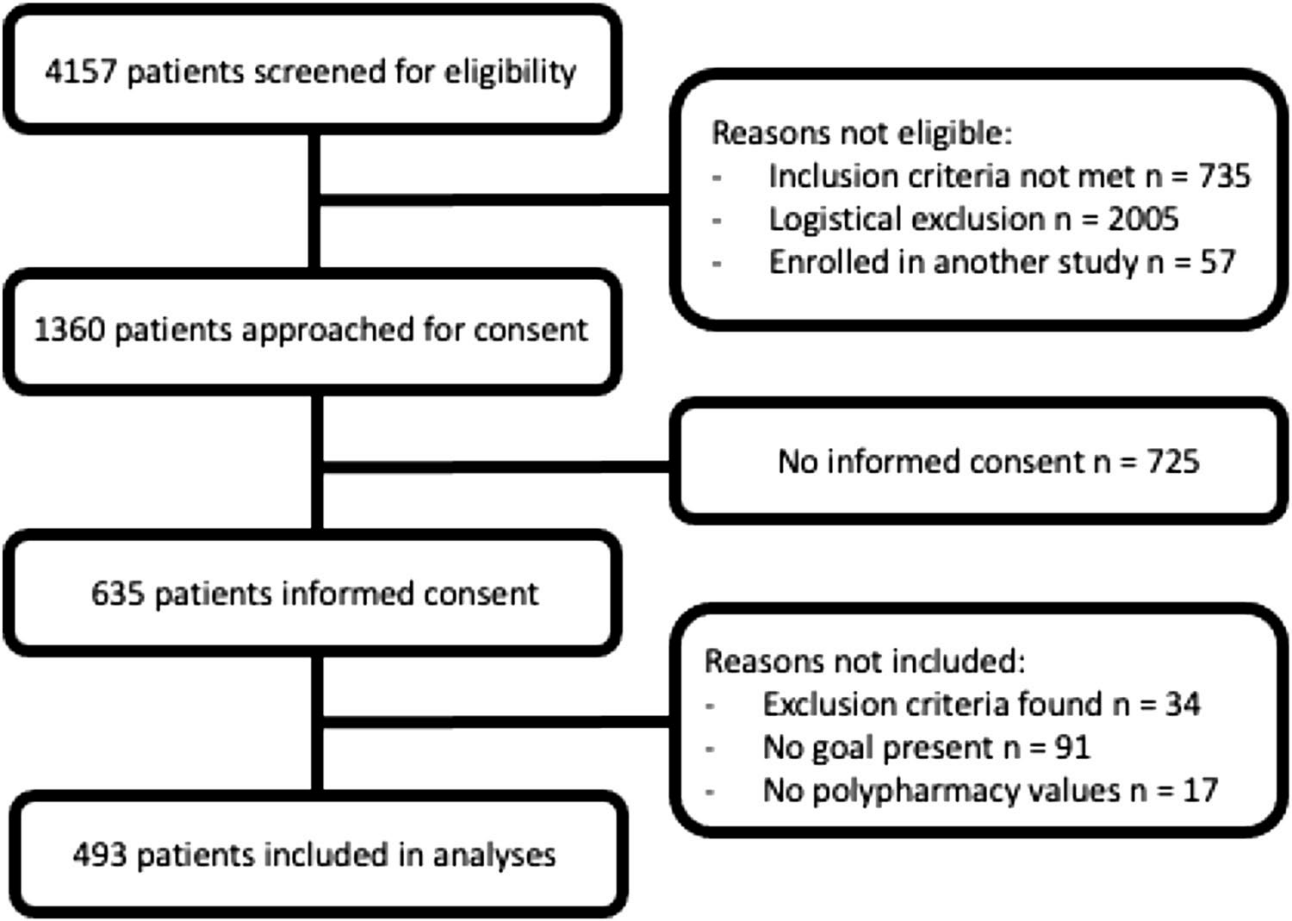
Flowchart of participant inclusion. Logistical exclusion reasons include that patients could not be reached within 96 h due to a transfer to another hospital, shortage of research staff (e.g., caused by National holidays) or absence of the patient in their room due to surgery or physical examination

Of 4157 screened patients, 1360 were eligible for inclusion of whom 635 (47%) gave informed consent. A total of 2005 patients were not approached for informed consent due to logistical exclusion reasons, which included patients who could not be reached within 96 h due to a transfer to another hospital, shortage of research staff (e.g., caused by National holidays) or absence of the patient in their room due to surgery or physical examination. In analyses, 493 patients were included (Fig. 1). The 91 patients who did not mention a goal (Appendix 1, Table 3) were less likely to have polypharmacy (41 vs 69%) and had higher scores on their self-rated health (69.4 vs 64.1) than patients who did mention a goal.

Demographic and geriatric characteristics of included patients (*n* = 493) can be found in Table 2. Of the 493 older hospitalized patients (median age 75 (IQR 72–80), 65% male), 223 patients presented with multimorbidity (45%). Patients were hospitalized for 7.9 ± 8.5 days on average and 65% of the patients with multimorbidity and 59% of the patients without multimorbidity were hospitalized acutely. Patients with multimorbidity experienced more mobility problems (67% vs 52%), and physical impairments (66% vs 50%) and rated their health lower (a score of 60.2 vs 67.5) compared to patients without multimorbidity.

**Table 2:** Demographic and geriatric characteristics describing patients included in the study (*n* = 493)

Demographics	Multimorbidity *n* = 223	No multimorbidity *n* = 270	*p*
Age (median (IQR))	75 (72–80)	75 (72–80)	0.70
Male	153 (69)	167 (62)	0.12
Living independent	211 (95)	261 (97)	0.26
Married	144 (66)	179 (65)	0.69
Education level			0.59
*Low*	67 (30)	81 (30)	
*Middle*	79 (38)	106 (39)	
*High*	77 (34)	83 (31)	
Acute admission	144 (65)	159 (59)	0.20

Geriatric assessment	Multimorbidity *n* = 223	No multimorbidity *n* = 270	*p*
Polypharmacy (≥ 5 medicines)	223 (100)	104 (39)	**< 0.001**
Comorbidity score (CCI) (median [IQR])	4 [3–5]	1 [1–2]	**< 0.001**
Fall last 6 months	75 (34)	73 (27)	0.11
Malnourished (SNAQ ≥ 2) (*n* = *488*)	45 (20)	45 (17)	0.34
Mobility problems (EQ-5D—mobility ≥ 1) (*n* = *490*)	150 (67)	138 (52)	**< 0.001**
Pain (EQ-5D—pain/complaints ≥ 1) (*n* = 492)	125 (56)	144 (54)	0.58
Self-rated health (EQ-5D-VAS) (mean ± SD) (*n* = *457*)	60.2 ± 18.2	67.5 ± 19.5	**< 0.001**
Physical impairment (Katz-15 ≥ 1) (*n* = *487*)	144 (66)	133 (50)	**0.001**
Cognitive impairment (6-CIT ≥ 8) (*n* = *400*)	19 (11)	25 (11)	0.96
Depression (PHQ-2 ≥ 1) (*n* = *481*)	88 (41)	85 (32)	0.06

All values are reported as number and percentage per patient group unless otherwise indicated. Boldface data are statistically significant

*CCI* Charlson comorbidity index, *SNAQ* Short Nutritional Assessment Questionnaire, *EQ-5D* EuroQol EQ-5D-3L, *EQ-5D-VAS* EuroQol EQ-5D Visual Analogue Scale (1–100), *Katz-15* Katz index of independence in activities of daily living with 15 questions on ADL and iADL, *6-CIT* Six-Item Cognitive Impairment Test, *PHQ-2* Patient Health Questionnaire-2

**Fig. 2:**
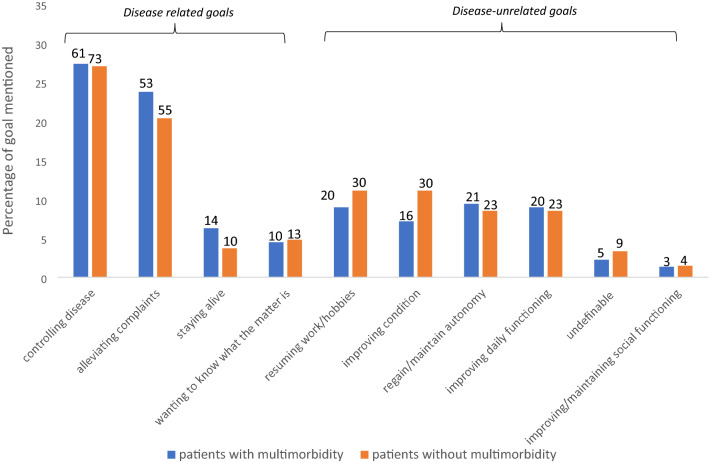
Visualization of goals mentioned by older hospitalized patients with (*n* = 223) and without (*n* = 270) multimorbidity displayed over the predefined categories. Goal categories have been subdivided in goals related to the disease patients are hospitalized for and disease-unrelated goals. Results have been displayed in percentage, to control for differences in group size. Absolute numbers are given above each bar

Overall, ‘controlling disease’ (27%) and ‘alleviating complaints’ (22%) were goals mentioned most by all older hospitalized patients (Fig. 2). After these two goals, patients with multimorbidity mentioned ‘regain/maintain autonomy’ (9%), while patients without multimorbidity mentioned ‘resuming work/hobbies’ (11%) and ‘improving condition’ (11%) equally often. Disease-unrelated goals were mentioned by 38% of patients with multimorbidity and by 44% of patients without multimorbidity.

**Fig. 3:**
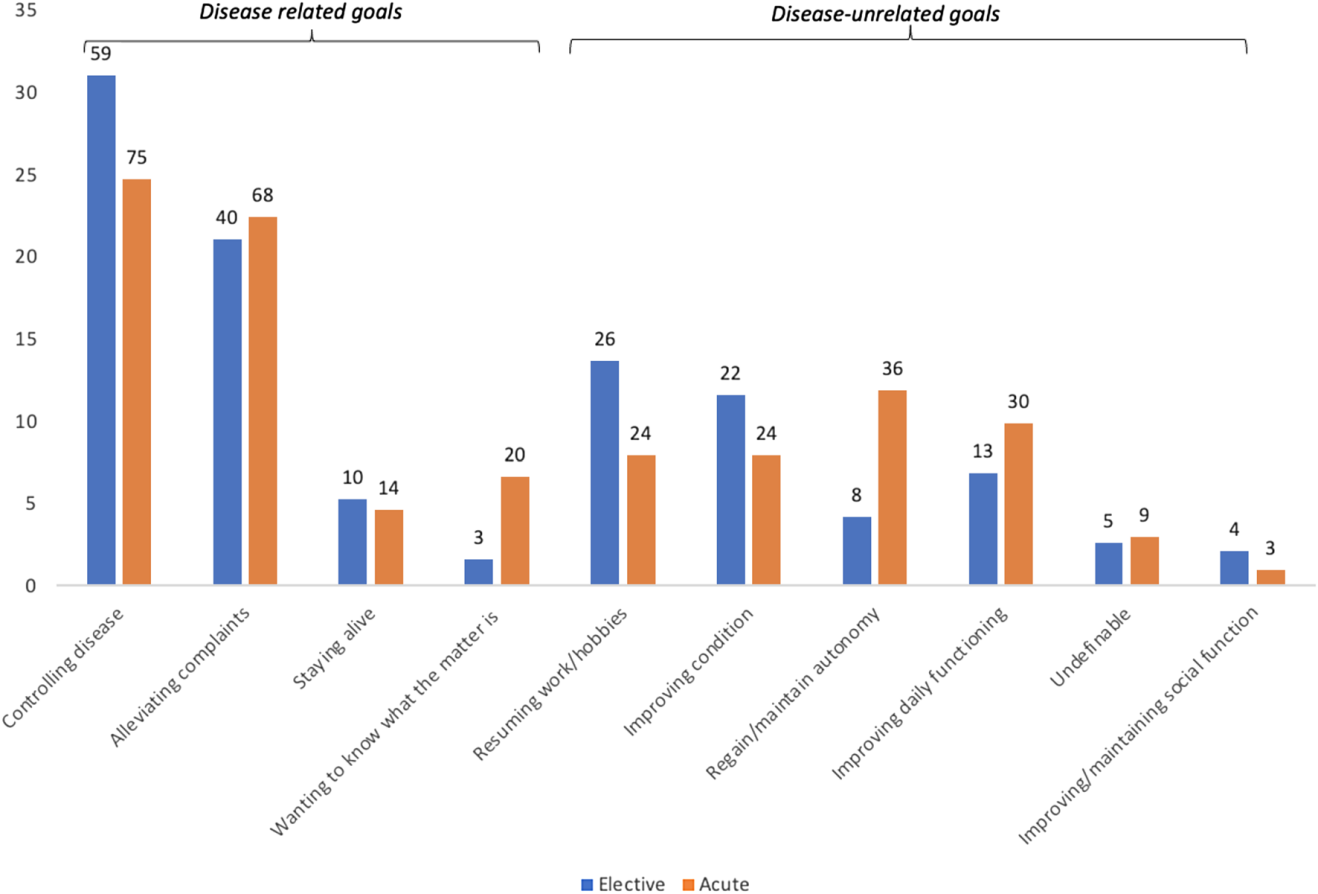
Visualization of goals mentioned by older patients that were in an elective (*n* = 190) and acute (*n* = 303) hospitalization displayed over the predefined categories. Goal categories have been subdivided in goals related to the disease patients are hospitalized for and disease-unrelated goals. Results have been displayed in percentage, to control for differences in group size. Absolute numbers are given above each bar

When dividing patients by their type of admission, either acute or elective (Fig. 3), both groups mentioned ‘controlling disease’ (31% and 25%) most often, followed again by ‘alleviating complaints’ (21% and 22%). Thereafter, patients with an elective admission mentioned ‘resuming work/hobbies’ (14%), while acutely admitted mentioned the goal ‘regain/maintain autonomy’ (12%). Disease-unrelated goals were mentioned by 41% of patients with an elective admission and by 42% of patients with an acute admission.

## Discussion

As far as we know, our study was the first to explore individual goals of older hospitalized patients with multimorbidity and compare them to goals mentioned by older hospitalized patients without multimorbidity. Goals mentioned most by all patients were ‘controlling disease’ and ‘alleviating complaints’. No major differences were found in goals mentioned between patients with and without multimorbidity. Both patients with and without multimorbidity mentioned more goals that were disease-related than disease-unrelated.

In line with a previously performed study in hospitalized older patients [18], we found ‘controlling disease’ and ‘alleviating complaints’ as goals mentioned most, independent of the presence of multimorbidity or type of admission. Finding this preponderance of disease-related goals could be expected. Being hospitalized brings you in an environment where the focus mostly lies on the disease the patient is hospitalized for [6, 29]. This focus is in line with the finding that older patients with multimorbidity see health problems as important, once they are severe, constant, uncontrolled or restrict daily activities [30].

However, our results also show that 41% of all older hospitalized patients mentioned disease-unrelated goals first, despite being hospitalized for an important health problem. From studies performed outside the hospital setting, we know older adults rate life enjoyment, social relations, quality of life, mobility and maintenance of autonomy as most important in daily life and the more chronic care setting [13–17]. Therefore, finding this substantial part in the hospitalized population as well, emphasizes the importance for healthcare professionals here to elicit individual patient goals to ensure healthcare is aligned with what the patient strives for.

Counterintuitive to our hypothesis, goals did not differ substantially between hospitalized older patients with and without multimorbidity. Asides from being in a disease-focused hospital environment, the disease burden experienced by the patient could have influenced our findings. When defining the presence of multimorbidity, we assumed that the group of patients with multimorbidity would experience more burden of their multiple conditions [1, 2]. However, patients with multimorbidity can become accustomed to their increased level of dependency, leading to reprioritization and a reduced possibility that the first goal mentioned is related to problems in daily life [31, 32]. For future research, it could, therefore, be interesting to measure subjective disease burden and assess if these are associated with goals of older hospitalized patients.

The population in our study was a large sample of older hospitalized patients who were admitted to various wards within the hospital (*n* = 493). However, some limitations were present in our study. The study was carried out within only one hospital, limiting its generalizability. A sample bias could have been present, as there was a male preponderance in our study (where in other studies there is a female preponderance) and 48% of the screened patients were not approached for informed consent due to logistical reasons. As, no gold standard exists to define multimorbidity [33], we made a combination of presence of two or more diseases and polypharmacy to increase certainty. Only the first mentioned goal by patients was analyzed. We assumed the first goal was the patients’ main goal, but this assumption could not be verified since we did not ask about the order of importance of mentioned goals. Patients’ goals were categorized into predefined categories, previously defined by Van der Kluit et al. [28] and these categories might not always be a good fit.

No major differences were found when comparing goals of older hospitalized patients with and without multimorbidity. More than a third of all patients mentioned goals that were disease-unrelated, emphasizing the importance for goal elicitation by healthcare professionals within hospital care to provide optimally integrated care.

## Data Availability

All data generated or analyzed during this study are included in this article. Further inquiries can be directed to the corresponding author.

## Appendix 1

See Table 3.

**Table 3:** Demographic and geriatric characteristics describing patients with and without a goal mentioned (*n* = 584)

Demographics	Goal mentioned *n* = 493	No goal mentioned *n* = 91	*p*
Age (median (IQR))	76 (72–80)	76 (72–80)	0.68
Male	320 (65)	59 (65)	0.99
Living independent	472 (96)	86 (95)	0.60
Married	323 (66)	57 (63)	0.60
Education level			0.72
*Low*	148 (30)	26 (29)	
*Middle*	185 (38)	32 (35)	
*High*	160 (33)	31 (34)	
Acute admission	294 (60)	54 (59)	0.84

Geriatric assessment	Goal mentioned *n* = 493	No goal mentioned *n* = 91	*p*
Polypharmacy (≥ 5 medicines)	336 (69)	37 (41)	**< 0.001**
Comorbidity score (CCI) (median [IQR])	2 [1–4]	2 [1–4]	0.49
Fall last 6 months	149 (30)	27 (30)	0.90
Malnourished (SNAQ ≥ 2) (*n* = *488*)	90 (18)	16 (18)	0.92
Mobility problems (EQ-5D—mobility ≥ 1) (*n* = *490*)	288 (58)	46 (51)	0.26
Pain (EQ-5D – pain/complaints ≥ 1) (n = 492)	269 (55)	47 (52)	0.83
Self-rated health (EQ-5D-VAS) (mean ± SD) (*n* = *457*)	64.1 ± 19.7	69.4 ± 18.9	**0.03**
Physical impairment (Katz-15 ≥ 1) (*n* = *487*)	277 (56)	44 (48)	0.38
Cognitive impairment (6-CIT ≥ 8) (*n* = *400*)	44 (9)	7 (8)	0.51
Depression (PHQ-2 ≥ 1) (*n* = *481*)	173 (35)	23 (25)	0.13

All values are reported as number and percentage per patient group unless otherwise indicated. Boldface data are statistically significant

*CCI* Charlson comorbidity index, *SNAQ* Short Nutritional Assessment Questionnaire, *EQ-5D* EuroQol EQ-5D-3L, *EQ-5D-VAS* EuroQol EQ-5D Visual Analogue Scale (1–100), *Katz-15* Katz index of independence in activities of daily living with 15 questions on ADL and iADL, *6-CIT* Six-Item Cognitive Impairment Test, *PHQ-2* Patient Health Questionnaire-2
